# Role of Netrin-1 Signaling in Nerve Regeneration

**DOI:** 10.3390/ijms18030491

**Published:** 2017-02-24

**Authors:** Xin-Peng Dun, David B. Parkinson

**Affiliations:** 1 Peninsula Schools of Medicine and Dentistry, Plymouth University, Plymouth, Devon PL6 8BU, UK; david.parkinson@plymouth.ac.uk; 2 School of Pharmacy, Hubei University of Science and Technology, Xianning 437100, China

**Keywords:** Netrin-1, DCC, Neogenin, Unc5A–D, bi-functionality, spinal cord, optic nerve, peripheral nerve, Schwann cells, regeneration

## Abstract

Netrin-1 was the first axon guidance molecule to be discovered in vertebrates and has a strong chemotropic function for axonal guidance, cell migration, morphogenesis and angiogenesis. It is a secreted axon guidance cue that can trigger attraction by binding to its canonical receptors Deleted in Colorectal Cancer (DCC) and Neogenin or repulsion through binding the DCC/Uncoordinated (Unc5) A–D receptor complex. The crystal structures of Netrin-1/receptor complexes have recently been revealed. These studies have provided a structure based explanation of Netrin-1 bi-functionality. Netrin-1 and its receptor are continuously expressed in the adult nervous system and are differentially regulated after nerve injury. In the adult spinal cord and optic nerve, Netrin-1 has been considered as an inhibitor that contributes to axon regeneration failure after injury. In the peripheral nervous system, Netrin-1 receptors are expressed in Schwann cells, the cell bodies of sensory neurons and the axons of both motor and sensory neurons. Netrin-1 is expressed in Schwann cells and its expression is up-regulated after peripheral nerve transection injury. Recent studies indicated that Netrin-1 plays a positive role in promoting peripheral nerve regeneration, Schwann cell proliferation and migration. Targeting of the Netrin-1 signaling pathway could develop novel therapeutic strategies to promote peripheral nerve regeneration and functional recovery.

## 1. Introduction

During nervous system development, growing axons produce a highly motile structure at their growing tips called the growth cone, which is required to detect environmental signals on their way to the correct targets. The environmental signals were first proposed by Cajal as “an intelligent force” based on his observations on commissural axons projecting toward the ventral midline in the chick embryonic spinal cord [1]. In Cajal’s earlier work, he envisioned both attractive and repulsive diffusible signals that determine the direction of axon projection [2]. However, in his later work, only attractive signals were proposed for axon guidance and the repulsive signals were not part of his hypothesis [3]. In 1963, the neuropsychologist Sperry proposed the chemoaffinity hypothesis, which stated that axons are guided by both attractive and repulsive signals [4]. However, Sperry’s chemoaffinity hypothesis was not widely accepted as a common mechanism for axonal guidance until the discoveries of four classic axon guidance family proteins: Netrin [5,6,7], Slit [8,9], Ephrin [10,11] and Semaphorin [12,13].

The importance of Netrins, Slits, Ephrins and Semaphorins for the formation of the mature nervous system is now well known, in particular, their ability to control precise axon targeting [14]. Among these four classic axon guidance family proteins, Netrin-1 possesses the strongest chemoattractive ability to promote axon extension [15]. Netrins, Slits, Ephrins and Semaphorins continue to be expressed in the adult nervous system and their expression is differentially regulated after nerve injury [16,17,18,19,20], but currently these classic axon guidance molecules have not been extensively studied for their role in nerve regeneration. Interestingly, both Cajal and Sperry studied axon regeneration in order to validate their hypotheses [3,4,21]. Cajal used adult rabbit regenerating sciatic nerve for his studies instead of embryonic tissue as the embryonic tissue did not allow him to obtain experimental evidence to prove his hypothesis [21]. In the early 1940s, Sperry performed a series of elegant experiments using the retinotectal system of the newt to obtain experimental evidence supporting his chemoaffinity hypothesis [4]. He sectioned the optic nerves and rotated the eyes 180 degrees to study how the newt restored their vision following the optic nerve injury. Sperry showed that these animals viewed the world “upside down” due to the 180-degree eye rotation [4,21]. These optic nerve regeneration studies led him to propose the chemoaffinity hypothesis [4]. Thus, Cajal and Sperry’s studies represent the earliest indications that axon guidance molecules could play a major role in axon regeneration [3,4,21]. However, research interests have not been focused on the role of classic axon guidance molecules in nerve regeneration due to embryonic or early post-natal lethality of most of the conventional gene knockout mice of these axon guidance molecules and their receptors, which has limited their use in studying the in vivo function of axon guidance molecules in adult nerve regeneration.

Unc6, the Netrin-1 homolog in *Caenorhabditis elegans* (*C. elegans*), was the first axon guidance molecule to be discovered among the four classic axon guidance family proteins and now Netrin-1 is one of the best characterized molecules regulating not only axonal guidance but also cell migration, morphogenesis and angiogenesis [15]. Netrin-1 is a bifunctional axon guidance cue attracting axons via the Deleted in Colorectal Cancer (DCC) receptor or Neogenin receptor and repelling axons via Uncoordinated receptor A–D (Unc5A–D) [15]. DCC, Neogenin and Unc5A–D all belong to the immunoglobulin (Ig) superfamily proteins [15]. Recently, Down Syndrome Cell Adhesion Molecule (DSCAM) and CD146 (also known as MCAM and Muc18) have also been identified as Netrin-1 receptors and both receptors belong to the immunoglobulin superfamily as well [22,23]. Other Netrin-1 receptors which do not belong to the immunoglobulin superfamily include heparan sulfate proteoglycans [24], α6β4 and α3β1 integrins [25,26] and, although somewhat controversial, the adenosine receptor (A2BR) [27]. Compared to its well characterized function for nervous system development, the potential for Netrin-1 function in nerve regeneration has not yet been fully studied. However, research interest about the role of Netrin-1 in nerve regeneration has been increasing in recent years. In the following sections of this article, we will review recent advances in Netrin-1 interaction with its immunoglobulin superfamily receptors and the available data for its role in spinal cord, optic nerve and peripheral nerve regeneration.

## 2. The Discovery of Netrin-1 and Its Immunoglobulin Superfamily Receptors

The search for axon guidance molecules began at the end of the 19th century when Cajal first proposed that diffusible cues may exist at the floor plate of the embryonic spinal cord to attract the projection of dorsal commissural axons toward the ventral midline [1]. However, there was no direct experimental evidence to show the existence of axon guidance molecules in the developing nervous system until 1986 with the demonstration that the extension of trigeminal sensory axons was attracted by the whisker pad epithelium, their final peripheral targets. The finding provided the first experimental evidence that chemoattractant signals existed during embryonic development [28]. The breakthroughs in the field of axon guidance were made in the early 1990s when the *Unc6* gene was cloned in *C. elegans* in 1992 [6] and then the Netrin-1 protein, the vertebrate ortholog of Unc6, was purified from chick brain in 1994 [7].

**Figure 1:**
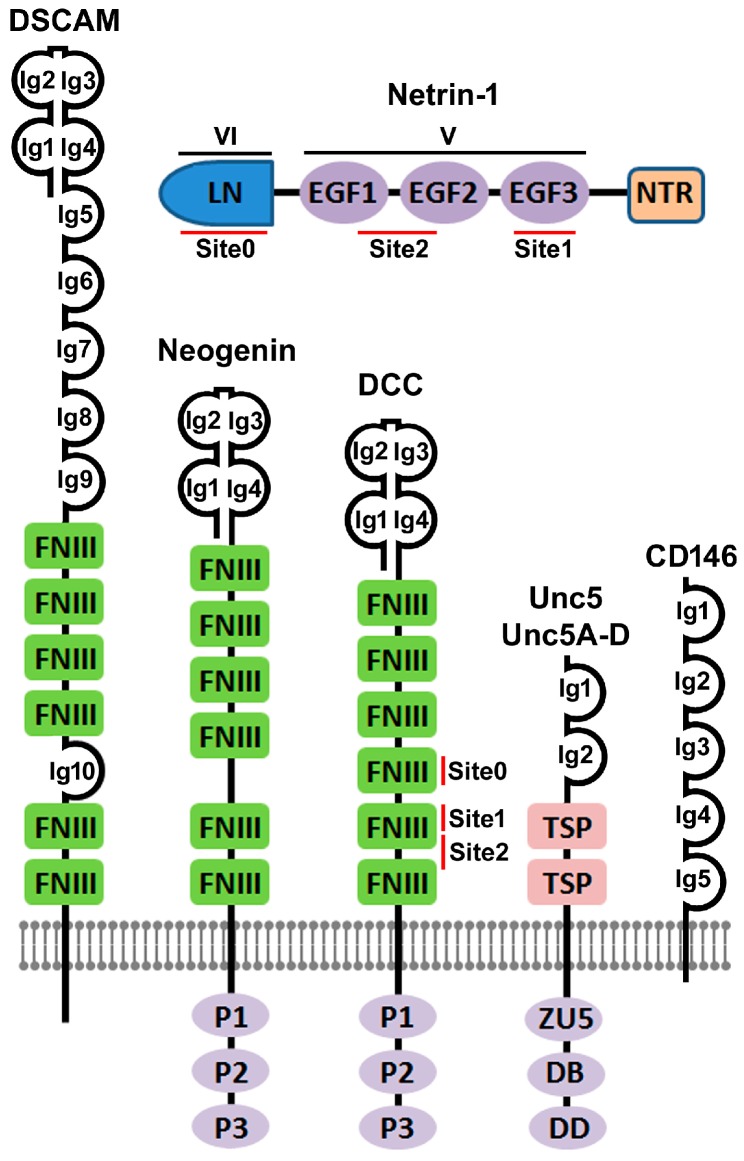
Structure of Netrin-1 and its immunoglobulin superfamily receptors. LN: laminin-like domain (also known as domain VI); EGF1-3: epidermal growth factor repeats (EGF1, EGF2 and EGF3 also known as domain V); NTR: C-terminal Netrin-like domain. The N-terminal four Ig domains (Ig1–4) in DSCAM and DCC form a horseshoe conformation that has been revealed by their crystal structure [35,36]. The horseshoe conformation in Neogenin is predicted [36]. CD146 also has five Ig domains but currently it is not known if it also has this horseshoe conformation. Ig: immunoglobulin; FNIII: fibronectin type III domain; TSP: thrombospondin type 1 (TSP-1) domain; ZU5: zona occludens 5 (ZU5) domain; DB: DCC-binding domain; DD: death domain [37].

Gene mutation studies reported in 1990 found that *Unc5*, *Unc6* and *Unc40* gene mutation resulted in an uncoordinated phenotype in *C. elegans* due to the disruptions of dorsal and ventral axon pathfinding and cell migration [5]. The *Unc6* gene encoded a signaling molecule that was required for the guidance of pioneer axons and the migration of cells along the body wall in *C. elegans* by interacting with Unc5 and Unc40 [5]. In 1992, the *Unc6* gene was cloned and the deduced amino acid sequence from the Unc6 cDNA showed that the *Unc6* gene encoded a 591 amino acid novel laminin-related protein [6]. In 1994, two vertebrate homologs of Unc6, named as Netrin-1 and Netrin-2, were identified from the explants of embryonic rat dorsal spinal cord and then purified from the chicken ventral neural tube tissue [7]. The amino acid sequences of chicken Netrin-1 and Netrin-2 were found to be 72% identical and they share about 50% identity with Unc6 protein. Netrin-1 and Netrin-2 are members of the laminin superfamily proteins and the N-terminal domains of Netrin-1 and Netrin-2 are most similar to the laminin gamma chain [15]. Netrin-1 and Netrin-2 consist of a laminin-like domain in the N-terminal (LN, also known as domain VI), followed by three epidermal growth factor (EGF) repeats (EGF1, EGF2 and EGF3, also known as domain V), and a C-terminal Netrin-like domain (NTR) [7,29] (Figure 1). Later, Netrin-3 [30], Netrin-4 [31], Netrin-5 [32], Netrin-G1 [33] and Netrin-G2 [34] have been identified in mammals. Thus far, Netrin-1 is the best characterized molecule among all the Netrin family proteins.

**Figure 2:**
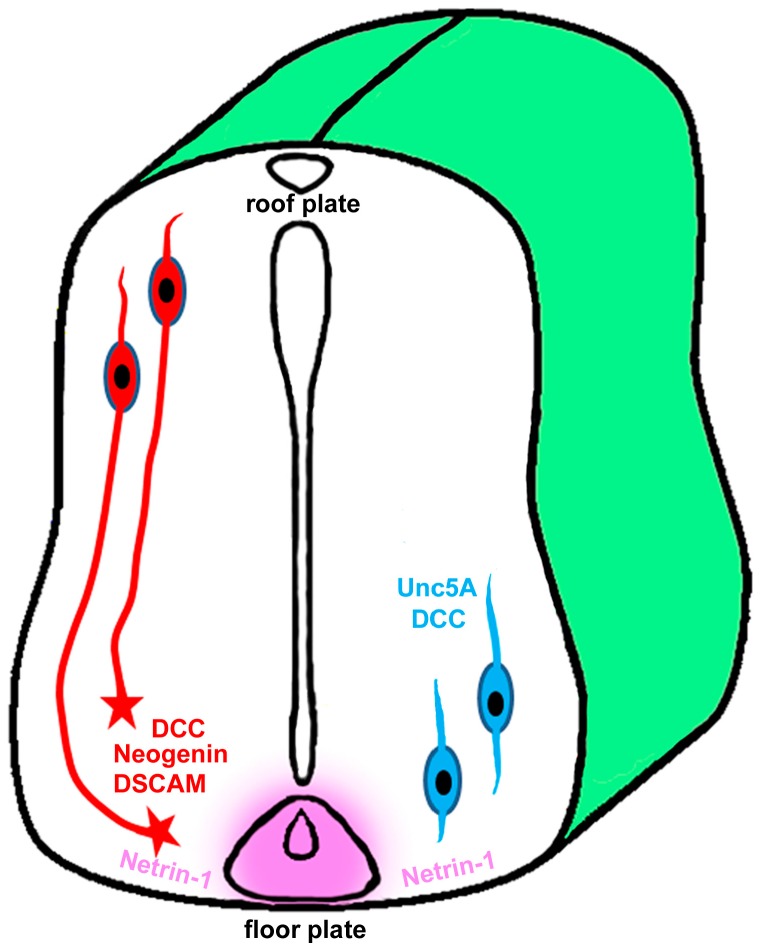
Netrin-1 functions in the developing spinal cord. Netrin-1 (pink) secreted by cells in the floor plate diffuses to the spinal cord tissue and forms a gradient concentration to attract dorsoventral projecting commissural axons (red) which express DCC, Neogenin or DSCAM. Oligodendrocyte precursor cells (blue) express both DCC and Unc5A [15] are repelled by Netrin-1 and migrate away from the ventral ventricular zone.

The *Unc40* gene was cloned in 1996 and functional assays showed that Unc40 was required in motile cells for movements towards as well as away from Unc6 sources [38]. In the same year, the *Drosophila* protein Frazzled was identified as another homolog of Unc40 [39]. Sequence analysis has revealed that the vertebrate DCC and Neogenin are closely related to Unc40, suggesting that DCC and Neogenin are Netrin-1 receptors in vertebrates. DCC was initially discovered as a tumor suppressor associated with an allelic deletion of chromosome 18 in human colorectal cancer [40] but later experimental studies have demonstrated that DCC mediates the chemoattractive guidance effect of Netrin-1 on rat spinal commissural axons (Figure 2) [41]. Neogenin was first identified in the chicken using a monoclonal antibody generated for isolating cell surface molecules from developing neural tissues. It was named as “Neogenin” because it is mainly expressed in cells that were undergoing terminal differentiation within newly generated tissues [42]. Experiments have demonstrated that Neogenin also mediates Netrin-1 attractive functions [15,41]. Both DCC and Neogenin are type I transmembrane receptors that belong to the immunoglobulin superfamily proteins [41]. Unc40, DCC, Neogenin and Frazzled all have a similar domain architecture containing four Ig domains at the N-terminus followed by six fibronectin type III (FNIII) domains, a single-pass transmembrane region and a large cytoplasmic tail that contains three highly conserved sequence motifs named as P1, P2 and P3 (Figure 1) [15]. P1, P2 and P3 motifs play a key role in downstream signal transduction upon Netrin-1 binding. The FNIII repeat number 4, 5 and 6 in both DCC and Neogenin are required for Netrin-1 binding [43,44].

The *Unc5* gene was first identified in *C. elegans* at the same time as the *Unc6* gene in 1990 [5] and its cDNA sequence was cloned in 1992 [45]. The *Unc5* gene encodes a 919 amino acid transmembrane protein and its extracellular N-terminus comprises two immunoglobulin domains [45]. The *Unc5* gene mutation disrupted the dorsal but neither ventral nor longitudinal cell migrations in *C. elegans*. Ectopic expression of Unc5 in Unc40 expressing neurons was sufficient to reorient the growth cone away from an Unc6 source suggesting that Unc5 acts as an Unc6 receptor to mediate Unc6 repulsive function [45,46]. Subsequently, four orthologs of *C. elegans* Unc5, Unc5A [47], Unc5B [47], Unc5C [48] and Unc5D [49], have been characterized in vertebrates. In line with the studies in *C. elegans*, Unc5A–D in vertebrates are required to mediate Netrin-1 repulsive signaling for axonal guidance and cell migration [15]. Unc5 and Unc5A–D are composed of two extracellular Ig domains followed by two extracellular TSP (thrombospondin) domains, a single-pass transmembrane region and an intracellular domain (Figure 1). The intracellular domain is made up of three conserved domains: a ZU5 domain (named after the mouse tight junction protein ZO-1 and *C. elegans* Unc5), a DCC-binding (DB) domain and a death domain (DD) (Figure 1). Although the Netrin-1 EGF2 domain binds to both Ig1 and Ig2 domains of Unc5 and Unc5A–D, the Ig1 domain is the primary interaction domain for Netrin-1 [50]. Extensive studies over the past twenty years have confirmed that the Netrin-1 repulsive signaling through Unc5 and Unc5A–D are highly conserved for axonal guidance and cell migration in *C. elegans*, *Drosophila melanogaster*, zebrafish, *Xenopus laevis*, chicken, rodents and humans [51].

DSCAM was originally identified as a gene that is duplicated in Down syndrome [52]. The *DSCAM* gene encodes a protein containing ten Ig domains and six FNIII repeats, a single-pass transmembrane region and an intracellular domain [52] (Figure 1). DSCAM is expressed by embryonic spinal commissural neurons in mammals and contributes to the guidance of these axons to the floor plate by functioning as a Netrin-1 receptor [22,53,54]. Soluble Netrin-1 binds to the surface of DSCAM-expressing cells with a similar affinity to that of Netrin-1/DCC interaction. DSCAM by itself, in the absence of DCC, is capable of mediating Netrin-1 signaling in activating phosphorylation of Fyn and Pak1 in DSCAM transfected cells. DSCAM Ig domains seven to nine were necessary and sufficient for Netrin-1 binding [22]. Netrin-1/DSCAM interaction initiates a chemoattractant response for spinal commissural axons but the interaction and signal transduction is independent of DCC [22]. Interestingly, DCC and DSCAM expression do not completely overlap each other in commissural neurons, suggesting that DSCAM is required to guide a different subset of commissural axons crossing the midline [53]. A recent study has revealed that DSCAM can form a receptor complex with Unc5C through extracellular domains to mediate Netrin-1 repulsive signaling [54].

A more recent study has reported that Netrin-1 binds to CD146 on endothelial cells with high affinity and that the Netrin-1/CD146 interaction in the vascular system promotes angiogenesis [23]. CD146 was originally identified in 1987 as a tumor marker for melanoma and is also known as Melanoma Cell Adhesion Molecule (MCAM or Mel-CAM) [55]. CD146 is a cell adhesion molecule and is also a member of the immunoglobulin superfamily [55]. Extensive studies showed that CD146 is involved in cell adhesion, migration, proliferation, differentiation and the immune response [56]. It contains five Ig domains in the extracellular fragment, a transmembrane region and a short cytoplasmic tail (Figure 1) [56]. Domain binding analysis revealed that the Netrin-1 EGF repeats bind to the extracellular Ig4 domain of CD146 [23]. CD146 is expressed in the spinal cord, dorsal root ganglia (DRG) and sciatic nerves in chicken embryos but its expression declines after hatching [57]. However, CD146 expression was upregulated in chicken regenerating sciatic nerves, DRG and dorsal horn of the spinal cord after sciatic nerve injury [57]. CD146 is strongly expressed in the rat nervous system during development but is weakly expressed in the adult rat brain [58]. Although a significant increase of CD146 expression in reactive astrocytes has been reported after rat hypoglossal nerve injury [59,60], it is currently unclear whether it plays a direct role in any regenerative response.

## 3. Crystal Structure Based Elucidation of Netrin-1 Bi-Functionality

One of the intriguing features of Netrin-1 is its bi-functionality for precise axon pathfinding. Netrin-1 binding to DCC alone leads to axon attraction [15]. The binding of Netrin-1 to the extracellular FNIII repeats of DCC brings DCC receptors together on the growth cone to form receptor clusters, enabling the cytoplasmic P3 motif of neighboring DCC receptors to interact with each other and recruit intracellular signaling complexes to activate the Src family kinase and trigger cytoskeleton rearrangement [61,62]. However, the Netrin-1 attraction is switched to repulsion when Unc5A–D is expressed with DCC in the same cell [37]. Netrin-1 binds to DCC and Unc5A–D though two different receptor binding sites and causes the association of DCC and Unc5A–D. This association enables the interaction between the DCC P1 motif and the DB domain of Unc5A–D to initiate Netrin-1 repulsive signaling [63]. 

Recent crystal structure studies have revealed that Netrin-1 has a rigid molecular architecture with little interdomain flexibility due to the formation of a disulfide bond network throughout the molecule as well as the short linkers between the individual Netrin-1 domains [43,44,50]. The crystal structure of chicken, human and mouse Netrin-1 (LN domain and three EGF repeats) show the same head-to-stalk arrangement structure in which the globular shaped LN domain forms the head and the three rod-like consecutive EGF repeats make up the stalk. It is an elongated molecule with a length of 150 Angstroms. The globular LN domain contains a conserved Ca^2+^ binding site and three glycosylation sites [43,44,50]. The Netrin-1 rigid molecular architecture requires Netrin-1 receptors to have more interdomain flexibility in order to undergo significant conformational changes for Netrin-1 binding [43,44,50].

**Figure 3:**
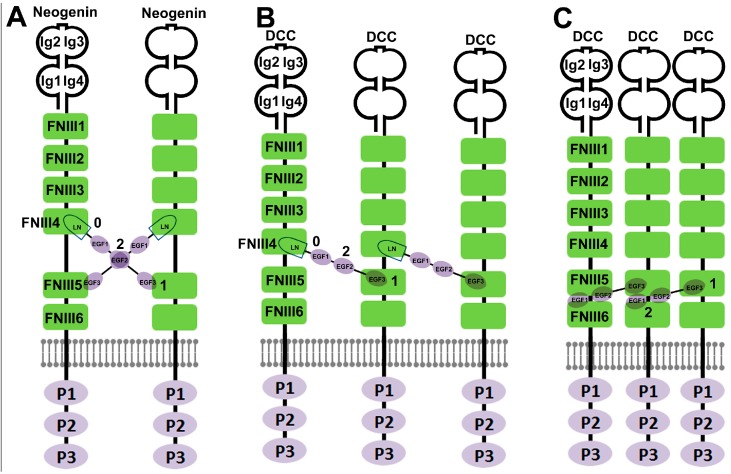
Schematic comparing the distinct models of Netrin-1/Neogenin and Netrin-1/DCC interaction. 0: site 0; 1: site 1; and 2: site 2. (**A**) Netrin-1 and Neogenin form a 2:2 ligand–receptor complex. Two Netrin-1 molecules form a head-to-head X-shaped dimer and interact with each other via the Netrin-1 EGF2 interfaces. (**B**) In Xu’s Netrin-1 and DCC binding model [43], the Netrin-1 LN domain interacts with the DCC FNIII4 domain, the Netrin-1 EGF3 domain interacts with the DCC FNIII5 domain, leading to Netrin-1 and DCC forming a continuous DCC-Netrin-1-DCC-Netrin-1-DCC assembly and the EGF2 domain of Netrin-1 is free for Unc5A–D binding. **(C**) In Finci’s Netrin-1 and DCC binding model [44], one Netrin-1 molecule still binds two DCC receptors but the EGF2 domain is engaged with the DCC FNIII5 and FNIII6 domains.

The Netrin-1/Neogenin and Netrin-1/DCC crystal structures were determined using chicken Netrin-1 (LN domain and EGF1-3 repeats) binding to mouse Neogenin or DCC (FNIII4 and FNIII5 domains) [43]. As revealed in the Netrin-1/Neogenin crystal structure, Netrin-1 does not undergo any significant conformational changes upon Neogenin binding due to its rigid molecular architecture. The Netrin-1 LN domain binds the Neogenin FNIII4 domain while the Netrin-1 EGF3 domain binds to the Neogenin FNIII5 domain. The distance between the two Neogenin binding sites on the Netrin-1 is almost twice as long as the distance between the two Netrin-1 binding sites on the Neogenin (Figure 3, site 0 and site 1). Therefore, the rigid Netrin-1 structure and the longer distance between two Neogenin binding sites on the Netrin-1 require two Neogenin receptors interacting with two different binding sites on Netrin-1. As a result, Netrin-1 and Neogenin form a 2:2 ligand–receptor complex (Figure 3). At the heart of the complex, two Netrin-1 molecules form a head-to-head X-shaped dimer and interact with each other via the Netrin-1 EGF2 interfaces. The X-shaped Netrin-1 dimer brings two Neogenin molecules together with Neogenin receptors arranged parallel to each other and their C-termini facing the same direction. In contrast, the Netrin-1/DCC protein complex shows a different overall architecture to the Netrin-1/Neogenin structure. Netrin-1 and DCC form a continuous DCC–Netrin-1–DCC–Netrin-1–DCC assembly but each Netrin-1 molecule still interacts with two DCC receptors via two different binding sites on the LN domain and the EGF3 domain (Figure 3). At the same time, each DCC receptor interacts with two Netrin-1 molecules via its two distinct Netrin-1 binding sites on the FNIII4 and FNIII5 domains [43]. Later, the binding site between the Netrin-1 LN and DCC FNIII4 domains was named as site 0 and the binding site between the Netrin-1 EGF3 and DCC FNIII5 domains was named as site 1 (Figure 1 and Figure 3) [64].

In the same year (2014) of the above report of the Netrin-1/Neogenin and Netrin-1/DCC crystal structures, the crystal structure of the human Netrin-1/DCC complex was reported using Netrin-1 binding to the DCC FNIII5 and FNIII6 domains [44]. In the Netrin-1/DCC (FNIII5 and FNIII6) complex, one Netrin-1 molecule also binds two DCC receptors (FNIII5 and FNIII6), however, a different binding site (named as site 2, Figure 1) has been identified in the Netrin-1/DCC (FNIII5 and FNIII6) crystal complex. At binding site 2, both EGF1 and EGF2 repeats interact with the DCC FNIII5 domain and the adjacent region of FNIII6 domain [44]. Netrin-1 site 2 contains only a few amino acid residues that directly interact with DCC. Mutagenesis of key amino acid residues at binding site 1 and site 2 demonstrated that both site 1 and site 2 are required for DCC mediated Netrin-1 attractive signaling but only site 2 is required for switching attractive signaling into repulsive signaling when Unc5 coexists with DCC in the same cell. Therefore, site 2 has been suggested as the key binding site for Netrin-1 bi-functionality [44]. In 2016, the mouse Netrin-1 crystal structure was reported [50]. Domain swapping and site mutagenesis for binding assays found that the Netrin-1 EGF2 domain interacts with Unc5B Ig1 and Ig2 domains. The key amino acid residues in the Netrin-1 EGF2 domain required for Unc5B binding are the same set of amino acid residues required for DCC binding [50]. Thus, site 2 is the key site responsible for switching Netrin-1 signaling from attraction into repulsion.

There are a high number of positively charged residues in binding site 2 of Netrin-1 and DCC [44]. Similarly, Unc5A–D Ig1 and Ig2 domains also contain groups of positively charged residues [65]. This suggested that negatively charged entities such as proteoglycans are required to be present in the interface of site 2 for Netrin-1 binding to DCC and Unc5A–D [65]. A previous study showed that the FNIII5 domain of DCC could bind heparin or heparan sulfates [66]. Further analysis by domain swapping and immunoprecipitation assays indicated that Netrin-1/heparin/DCC or Netrin-1/heparin/Unc5 ternary complexes could form at site 2 [65]. Furthermore, Netrin-1-mediated commissural axon guidance requires cell-autonomous expression of heparan sulfate in commissural neurons [67]. These findings support the idea that the property of a heparan sulfate molecule presenting at site 2 could also determine Netrin-1 bi-functionality. Heparan sulfate molecules in favor of DCC binding initiate chemoattraction but heparan sulfate molecules in favor of Unc5A–D binding will switch chemoattraction into chemorepulsion [44].

Based on the above findings, the molecular mechanism of Netrin-1 bi-functionality could be envisioned. Firstly, the rigid Netrin-1 structure and the long distance between different receptor binding sites on Netrin-1 allows one Netrin-1 molecule to bind at least two receptors. This enables Netrin-1 to cross-link with different receptor types via different binding sites to initiate distinct functions. Secondly, the highly positive charged patches on site 2 offer adaptability for Netrin-1 to accommodate different receptor types such as DCC, DSCAM and Unc5A–D. Thirdly, DCC- or Unc5A–D-selective negatively charged molecules such as heparan sulfates could also determine Netrin-1 attractive or repulsive signaling.

However, the question remains of how Netrin-1 receptors interact with Netrin-1 at site 2. In the Netrin-1/DCC (FNIII4 and FNIII5) binding model [43], the Netrin-1 site 2 is freely available for Unc5A–D binding even when site 0 and site 1 are engaged with DCC receptors. In contrast, in the Netrin-1/DCC (FNIII5 and FNIII6) binding model [44], a two-stage binding model has been suggested. First, site 2 binds to a DCC receptor and then Unc5A–D replaces it when a Netrin-1 repulsive signaling is required. Thus, the Netrin-1/DCC binding model needs to be further examined by studying the Netrin-1/DCC (FNIII4–6 domains) crystal structure. Furthermore, an anti-Netrin-1 antibody that binds to the Netrin-1 EGF2 domain has been generated. This antibody could block Netrin-1/Unc5B interaction to inhibit Netrin-1 induced tumor cell growth but does not inhibit Netrin-1/DCC interaction, suggesting that DCC and Unc5B also could interact with different Netrin-1 motifs in the EGF2 domain. Moreover, it is currently not clear how site 0 and site 1 interact with DCC when site 2 is engaged with Unc5A–D. Finally, in the Netrin-1/Neogenin crystal complex, two Netrin-1 molecules interact with each other via their EGF2 domain interface and form a head-to-head X-shaped dimer to stabilize the Netrin-1/Neogenin protein complex. The Netrin-1 dimer formation will require a different molecular mechanism for switching the Neogenin attractive signaling to repulsive signaling because site 2 in Netrin-1/Neogenin complex is masked by Netrin-1 itself, unlike site 2 in Netrin-1/DCC complex which is freely available or masked by a DCC receptor. In fact, the repulsive signaling through Neogenin is largely initiated by the repulsive guidance molecules (RGMs), another family of ligands for the Neogenin receptor, rather than Netrin-1 [68]. 

Netrin-1 has bi-functionality not only on axonal guidance and cell migration but also on angiogenesis [23,69,70]. However, it was recently reported that the pro-angiogenic effect of Netrin-1 is mediated by the CD146 receptor, which lacks the FNIII repeats in its extracellular domain (Figure 1) [23]. Unc5B is also highly expressed in endothelial cells in the vascular system and has been suggested to mediate Netrin-1 anti-angiogenic activity [70]. The Netrin-1 EGF2 domain binds to Unc5B Ig1 and Ig2 domains [50]. Netrin-1 also binds to the CD146 Ig4 domain though its EGF repeats [23] but which EGF repeat is required for CD146 binding has not been further examined. Currently, it is not clear if Unc5B can form heterodimers with CD146 through Netrin-1 binding in order to switch the Netrin-1 pro-angiogenic effect to an anti-angiogenic effect. Netrin-1 directly binds CD146 with high affinity (Kd = 1.3 nM) which is higher than its affinity for Unc5B (Kd = 5.1 nM). Netrin-1 treatment of endothelial cells resulted in a biphasic response with low doses (50–200 ng/mL) inducing proliferation, migration and tube formation and high doses (1000–2000 ng/mL) inhibiting these effects. Knockdown experiments showed that the low dose effect is mediated by the CD146 receptor, whereas the high dose effect is through Unc5B signaling [23]. Other studies also showed that Netrin-1 bi-functionality in the vascular system is dose dependent [69,70]. Thus, in the vascular system, Netrin-1 bi-functionality could be also determined by the Netrin-1 concentration, although the high dose effect will not happen in normal Netrin-1 physiological concentrations, this could perhaps occur in experimental studies or pathological conditions.

## 4. Role of Netrin-1 in Spinal Cord Injury and Repair

Netrin-1 shows a consistent level of expression in the rodent spinal cord throughout the embryonic stage to adulthood [17]. In the adult rat spinal cord, Netrin-1 is expressed in the white matter, all lamina of the grey matter, cells of the central canal and the meninges [17,71]. Oligodendrocytes but not astrocytes express Netrin-1 in both the white and the grey matter of the adult rat spinal cord [17,71]. In the grey matter, Netrin-1 is highly expressed by multiple classes of spinal interneurons in the dorsal horn. Cell bodies and processes of motor neurons in the ventral horn also express Netrin-1 but the level is lower than dorsal interneurons [17]. Unlike the developing nervous system where Netrin-1 largely acts as a long range diffusible signal, Netrin-1 protein is enriched in periaxonal myelin membranes in the white matter of adult spinal cord [17]. The membrane-associated Netrin-1 indicates that, in the mature nervous system, Netrin-1 may act as a short-range cue to maintain appropriate central nervous system (CNS) function. After spinal cord injury, Netrin-1 is still expressed by neurons and oligodendrocytes immediately adjacent to the lesion and the level is maintained in neurons and oligodendrocytes outside the area of lesion [71,72]. However, inside the lesion, Netrin-1 mRNA and protein levels were dramatically reduced in neurons and oligodendrocytes. Netrin-1 down-regulation at the injury site persists at least seven months after spinal cord injury [72].

Netrin-1 receptors, DCC, Neogenin and Unc5A–D all are continuously expressed in the adult rat spinal cord [72,73,74,75,76,77]. DCC and Neogenin are highly expressed in the embryonic spinal cord, but the relative amounts of DCC and Neogenin proteins decrease gradually during spinal cord maturation. DCC and Neogenin are still detectable in adult rat spinal cord although their expression is very low [74]. In adult rat spinal cord, DCC and Neogenin are largely expressed by neurons in the ventral horn of the spinal cord and their expression is very low in the dorsal horn of spinal cord [74]. In contrast, the expression of Unc5A–C is increased in the adult rat spinal cord compared to their levels in the embryonic spinal cord [74]. The expression level of Unc5D is substantially lower than Unc5A–C in the adult rat spinal cord [74,75]. Unc5A is expressed throughout all laminas of the gray matter except laminas I, II and IX [71,74]. Unc5B is not only highly expressed by neurons throughout all laminas of the gray matter but also is highly expressed by glial cells in the white matter [74]. Unc5C is predominantly expressed in lamina II and IX of the gray matter [71,74].

The cell-specific expression pattern of Netrin-1 receptors is maintained after spinal cord injury but the levels of Netrin-1 receptor expression were reduced in the lesion area as well as in the adjacent rostral and caudal spinal cord [71,72,76]. One month after injury, DCC mRNA expression is reduced to 80% in the adjacent rostral spinal cord and 66% in the adjacent caudal spinal cord [71]. Similar to the Netrin-1 down-regulation, DCC protein at the site of injury remained at ~50% of pre-injury levels even seven months after injury [72]. Unc5A–D mRNAs are all down-regulated after injury in the lesion area as well as in spinal cord segments adjacent to the lesion site [71,72,76]. One month after injury, Unc5A mRNA is reduced to 43% in the adjacent rostral spinal cord and 30% in the adjacent caudal spinal cord. Unc5C mRNA is reduced to 59% in the adjacent rostral spinal cord and 45% in the adjacent caudal spinal cord at the same timepoint [71,76]. Unc5A–C proteins were down-regulated in the lesion site from Day 3 onwards and the down-regulation was maintained until one month following injury, but there were no significant differences 40 days following injury compared to the protein levels in intact spinal cord [72]. Recently, Neogenin down-regulation has been reported in the adult lamprey after spinal cord injury [78]. 

The expression pattern of Netrin-1 and Netrin-1 receptors in adult spinal cord and their expression changes after spinal cord injury are summarized in Table 1. The post-injury distribution of Netrin-1 protein in the spinal cord is similar to other myelin associated inhibitors of axonal regeneration, such as Nogo, myelin associated glycoprotein, Semaphorin and Ephrin [79,80]. The dominant expression of Unc5A–C in the spinal cord after injury and the persistent expression of Netrin-1 by oligodendrocytes surrounding the lesion support a hypothesis that Netrin-1 is a myelin-associated inhibitor of axonal regeneration after spinal cord injury. In vitro studies showed that neutralization of Netrin-1 in myelin prepared from adult rat spinal cord increased neurite outgrowth from Unc5A–D expressing spinal motor neurons [71]. In vivo grafting of Netrin-1 overexpressing fibroblasts into the dorsal column lesion cavity also demonstrated that Netrin-1 inhibits axonal regeneration [71]. Therefore, Netrin-1 has been considered as an oligodendrocyte-associated inhibitor that contributes to axonal growth failure after adult spinal cord injury.

## 5. Role of Netrin-1 in Optic Nerve Development and Regeneration

Retinal ganglion cells (RGCs) are the only retinal neurons to project from the periphery into the brain and their peripheral location makes the optic nerve a convenient system for studying CNS regeneration. During optic nerve development, newly generated RGCs project their axons through the optic disc and then bundle together to form the optic nerves [81]. Netrin-1 is expressed in the optic nerve head and is required to guide visual axons out of the eye [82]. Here, Netrin-1 acts as a short-range guidance cue to attract RGC growth cones into the optic nerve head. In Netrin-1 deficient mice, RGC axons could reach the optic disc but failed to enter the optic nerve head and instead grew aberrantly in other regions of the retina [82]. The Netrin-1 attractive effect is mediated by the DCC receptor, which is expressed on the growth cones of RGCs and DCC knockout mice exhibit similar retinal abnormalities as the Netrin-1 knockout mice [82,83]. Netrin-1 is further expressed along the pathway of the optic nerve, but the Netrin-1 expression pattern further down the nerve indicated that Netrin-1 functions later on as a repellent to keep the RGC axons inside the optic nerve path [84]. Consistent with this idea, in vitro assays showed that the response of RGC axons to a gradient of Netrin-1 depends on the developmental stages of RGC axons [84]. Early stage axons that have not yet reached the optic nerve head show attraction towards a source of Netrin-1 but RGC axons that have grown through the optic nerve head are insensitive to Netrin-1. However, RGC axons that have crossed the optic chiasm are strongly repelled by Netrin-1 [84]. These results support the idea that Netrin-1 functions as a repellent in the distal part of the visual pathway and helps to constrain the projecting RGC axons inside the optic nerve trajectory. DCC protein was no longer detectable on the distal axonal segments when the majority of RGC axons had projected through the optic disc despite significant DCC protein expression on the proximal axonal membranes in the nerve fiber layer [83]. Furthermore, RGC axons do turn on the expression of Unc5A and Unc5B in the later stage of optic nerve projection [85,86]. This could explain why Netrin-1 has the age-related response during optic nerve development [81].

Many axon guidance molecules are down-regulated with the maturation of the visual system in mammals [87]. However, Netrin-1, DCC, Unc5A and Unc5B are found to be continuously expressed in adult rat RGCs [85,86]. In addition to the expression in RGCs, Netrin-1 is also expressed by glial cells in the optic nerve [86]. After rat optic nerve injury, DCC, Unc5A and Unc5B were all down-regulated in RGCs [85,86]. Examining DCC and Unc5B expression in RGCs after rat optic nerve transection and repair using grafted sciatic nerve revealed that both DCC and Unc5B were still down-regulated in RGCs even with the sciatic nerve graft [86]. Unlike mammals, the optic nerve in goldfish can successfully regenerate following injury and several studies showed that the developmental guidance cues are completely re-expressed in the adult goldfish visual system after optic nerve injury [88,89,90]. Interestingly, the expression of Netrin-1 is maintained at the adult goldfish optic disc and Netrin-1 receptors are up-regulated in RGCs after optic nerve injury [85].

The optic nerve belongs to the CNS and regeneration is often limited after injury [85,86,87,88,89,90]. The Netrin-1 and Netrin-1 receptor expression pattern in the adult rat visual system and their injury response are very similar to their expression pattern and injury response in the adult rodent spinal cord. Although RGC cell bodies expressed DCC, DCC protein is absent in the RGC regenerating axons [85]. Thus, Netrin-1 could function as an inhibitor during optic nerve regeneration but this remains to be investigated. 

## 6. Netrin-1 and Netrin-1 Receptors Expression in Intact and Injured Peripheral Nerves

Netrin-1 mRNA and protein are expressed in the adult rat sciatic nerve and Schwann cells are the major cell type to secrete Netrin-1 [16,86,91]. Netrin-1 mRNA levels did not change in the rat distal nerve stump two weeks following sciatic nerve crush injury but increased 40-fold two weeks after sciatic nerve transection and immediate anastamosis. The dramatic increase of Netrin-1 expression was mainly produced by Schwann cells [16]. Netrin-1 protein expression was up-regulated in the distal nerve stump at 3, 7 and 14 days after rat sciatic nerve transection injury [73]. Netrin-1 is also expressed in the rat dorsal root ganglia (DRG) but there was no significant change of expression after sciatic nerve injury [91]. The mouse median nerve also expresses Netrin-1 [92]. There was no significant change of Netrin-1 mRNA expression in the nerve segment distal to the injury site seven days after mouse median nerve transection and repair, but Netrin-1 mRNA increased 1.9-fold and 2.4-fold two weeks and three weeks respectively following mouse median nerve transection and immediate anastamosis [92]. Netrin-1 protein was also significantly up-regulated in the median nerve segment distal to the injury site at Day 14 [92]. In the zebrafish, Netrin-1b mRNA has been reported to be expressed by Schwann cells along the motor nerve both before and after motor nerve transection injury [93]. Thus, the available data indicated that Netrin-1 is expressed in the Schwann cells of the intact peripheral nerves and it is upregulated in Schwann cells of the distal nerve segment after peripheral nerve transection injury.

Peripheral nerve injury damages two major populations of axons, motor axons whose cell bodies localize in the ventral horn of the spinal cord and sensory axons, the cell bodies of which localize in the DRG. We have already reviewed Netrin-1 receptor expression in adult rodent spinal cord in Section 4 above. DCC, Neogenin and Unc5A–D are all expressed in the motor neurons localized to the ventral horn of the adult rodent spinal cord [72,73,74,75,76]. Thus far, there is no report available to examine the expression changes of Netrin-1 receptors in motor neurons after peripheral nerve injury. Similar to the expression levels of Netrin-1 receptor in adult rat motor neurons, adult rat DRG neurons express high levels of Unc5A–C and low levels of DCC and Unc5D [75,91]. DCC expression was significantly up-regulated in the DRG at seven days following sciatic nerve injury, but both Unc5B and Unc5C were down-regulated [91]. Unc5B protein was expressed in both intact and injured rodent sciatic nerve [73,94]. Immunostaining indicated that Schwann cells in the sciatic nerve express Unc5B both before and after sciatic nerve injury [91]. Cultured Schwann cells express Netrin-1, Neogenin, Unc5A and Unc5B, but the expression of Unc5A mRNA is very low compared to the level of Unc5B and Neogenin expression [73,95].

Due to the limited information on Netrin-1 receptor expression in the peripheral nervous system, in particular, any changes of expression in response to peripheral nerve injury, we have analyzed three published microarray data sets (GSE30165 [96,97], GSE22291 [98] and GSE74087 [99]) after sciatic nerve injury to assess Netrin-1 receptor expression in the intact rodent peripheral nervous system and any expression changes after peripheral nerve injury. The GSE30165 data set studied the time course of the gene expression profile in adult rat DRG (L4 and L5) and the proximal nerve segment after 0, 1, 4, 7 and 14 days following sciatic nerve transection injury [96,97]. Analyzing the GSE30165 data set showed that, in the intact rat peripheral nervous system, the expression levels of Neogenin and Unc5A–C are similar between sciatic nerve and DRG samples but DCC and Unc5D have much lower expression values in sciatic nerve compared to the DRG. After sciatic nerve injury, DCC and Unc5B–D are down-regulated while Unc5A is up-regulated in the DRG; the Neogenin mRNA level is unchanged in the DRG after sciatic nerve injury. 

A similar expression pattern and injury response was revealed for all the Netrin-1 receptors between data set GSE22291 and data set GSE74087 which studied the gene expression profile in the mouse distal nerve stump at 3, 7 and 14 days after sciatic nerve crush and transection injury respectively. DCC, Unc5A and Unc5D mRNA showed no significant changes after sciatic nerve injury while Neogenin mRNA is significantly down-regulated on Day 3. Unc5B mRNA is up-regulated on both Day 3 and Day 7. Unc5C mRNA is down-regulated at Day 3 and then up-regulated at Day 7. 

## 7. Netrin-1 Regulates Schwann Cell Proliferation and Migration

In response to injury, Schwann cells first shed their myelin and then proliferate to form the Bands of Büngner in the distal nerve stump to direct regenerating axons to their targets. In the case of a transection injury where a nerve gap is generated, Schwann cells migrate into the nerve gap to form a nerve bridge together with other cell types. This newly formed nerve bridge acts as substrate for axons navigating across the peripheral nerve gap. Interestingly, two studies indicate that Netrin-1 could promote cultured Schwann cell proliferation and migration [73,95].

Netrin-1 treatment (50 ng/mL) induced RT4 schwannoma cell proliferation [73]. An examination of Netrin-1 receptor mRNA expression using RT-PCR in RT4 schwannoma cells and primary rat Schwann cells found that RT4 schwannoma cells and rat Schwann cells strongly expressed Unc5B. Knockdown Unc5B in RT4 schwannoma cell using Unc5B specific RNAi significantly reduced Netrin-1 induced RT4 schwannoma cell proliferation [73]. Therefore, the effect of Netrin-1 on Schwann cell proliferation has been suggested to be mediated by the Unc5B receptor [73]. Another report using RSC96 cells (an immortalized rat Schwann cell line) studied the effects of Netrin-1 upon Schwann cell proliferation and migration [95]. RSC96 cells expressed very low levels of Unc5A, a slightly higher level of Neogenin and high levels of Unc5B. Netrin-1 showed no effect on RSC96 cell proliferation with a series of Netrin-1 concentrations ranging from 10 ng/mL to 500 ng/mL. Instead, Netrin-1 regulated RSC96 Schwann cell migration through the activation of p38 mitogen activated protein kinase (MAPK) and PI3K-Akt. At 100 ng/mL concentration, Netrin-1 increased p38 MAPK and Akt phosphorylation and promoted RSC96 Schwann cell migration. At 500 ng/mL concentration, Netrin-1 decreased p38 and Akt phosphorylation and inhibited RSC96 Schwann cell migration. The effect of Netrin-1 on Schwann cell migration has been suggested to be mediated by the Unc5B receptor due to Unc5B having the highest expression level in RSC96 Schwann cells compared to the expression levels of Neogenin and Unc5A [95].

Previous work has shown that Netrin-1 dose-dependently regulates endothelial cell proliferation and migration through extracellular signal-regulated kinase (ERK) and p38 MAPK signaling pathways [23,100]. At a concentration from 100 ng/mL to 200 ng/mL, Netrin-1 increases p38 and ERK phosphorylation and subsequently promotes endothelial cell proliferation and migration [23,100]. At a concentration of 1000 ng/mL or higher, Netrin-1 decreases p38 and ERK phosphorylation and subsequently inhibits endothelial cell proliferation and migration [23]. The Netrin-1 attractive function on endothelial cells is mediated by the CD146 receptor and its inhibitory function is mediated by the Unc5B receptor [23,70,101]. Our recent work has found that Schwann cell express high level of CD146 (unpublished data), thus, it is possible that the Netrin-1 induced Schwann cell proliferation and migration at low concentration is mediated by CD146 and its inhibitory effect at high concentration is mediated by the Unc5B receptor. Using a single cell migration assay based on time-lapse imaging, Netrin-1 showed no effect on Schwann cell migration [56]. In this study, the pipette tip was loaded with 50 µg/mL Netrin-1 which presented a gradient of Netrin-1 concentration to the cultured rat primary Schwann cells. This concentration, however, was much higher than the Netrin-1 low dose effect on RSC96 cells and endothelial cells described above. It is usually thought that the Netrin-1 physiological concentration ranges from 50 ng/mL to 150 ng/mL since it was first measured in chicken [7,100,102]. This physiological concentration supports the above findings that Netrin-1 could promote Schwann cell proliferation and migration in vivo following peripheral nerve injury but this remains to be investigated.

## 8. Netrin-1 Signaling in Peripheral Nerve Regeneration

In Section 6 above, we discussed DCC, Neogenin and Unc5A–D expression in adult DRG neurons. During development, DRG neurons extend their axons toward the dorsolateral part of the spinal cord and enter the spinal cord through the dorsal root entry zone (DREZ) around Embryonic Day 12. At this stage, when DRG axons orient themselves toward the DREZ, Netrin-1 is expressed in the dorsal spinal cord to time the entry of DRG axons [103]. At the same time, Netrin-1 is expressed in the ventral spinal cord to prevent DRG axons from projecting aberrantly towards this area [104]. Here Netrin-1 acts as a repulsive guidance cue for sensory axons projecting toward the spinal cord and Unc5C has been suggested as the Netrin-1 receptor mediating this effect [103,104]. Adult sensory neurons express high levels of Unc5A–C and Netrin-1 treatment (500 ng/mL) inhibited neurite outgrowth in both adult DRG explants and dissociated DRG neuron cultures, therefore, Netrin-1 has been suggested as a repellent for regenerating sensory axons [75]. However, a high concentration of Netrin-1 (500 ng/mL) has been used to treat DRG explants and dissociated DRG cultures in the study. As discussed above for the Netrin-1 high dose effect on endothelial cell and Schwann cell proliferation and migration, it is not surprising that Netrin-1 at a concentration of 500 ng/mL could inhibit neurite outgrowth of sensory neurons because adult sensory neurons express high level of Unc5A–C [73,75].

It is well known that a preconditioning lesion can reprogram adult DRG neurons and promote adult sensory neuron regeneration into the spinal cord [105]. Furthermore, it has been shown that DCC mRNA was upregulated while Unc5B and Unc5C mRNA were down-regulated in sensory neurons after rat sciatic nerve transection [91]. Therefore, the effect of Netrin-1 has been tested on pre-conditionally lesioned DRG cultures but Netrin-1 treatment (5 ng/mL or 200 ng/mL) did not alter the overall outgrowth of DRG neurite length or number of branches per process. To further evaluate the role of Netrin-1 signaling in nerve regeneration, siRNA knockdown of either DCC or Unc5B in sensory neurons has been performed by continuous injection of DCC or Unc5B siRNA at the injury site. Seven days after continuous DCC or Unc5B siRNA injection, regenerating nerve stumps exposed to DCC siRNA had fewer axons extending into the injury site as well as a decrease in the total number of regenerating fibers. In contrast, administering Unc5B siRNA to the regenerating nerve front significantly increased the distal extension of activated Schwann cells and the axons across the injury site. Taken together, these data indicated that the DCC receptor facilitates while the Unc5B receptor hinders peripheral axon regeneration. Based on these observations, Netrin-1 signaling has been suggested to play an important role in the regulation of in vivo peripheral nerve regeneration. Exogenous Netrin-1 protein at a concentration of 100 µg/mL has been injected to the injury site of rat sciatic nerve to test if an increase in Netrin-1 concentration could promote axonal growth in vivo, but Netrin-1 at this concentration did not alter in vivo either Schwann cell migration or axon extension. However, the low dose effect of exogenous Netrin-1 has not been tested yet. A more recent report showed that transplanting Netrin-1 overexpressing bone marrow mesenchymal stem cells to the injury site of rat sciatic nerve promotes axon regeneration and functional recovery [106]. Thus, the DCC and Unc5B knockdown data together with the data from Netrin-1 overexpressing stem cells suggests that Netrin-1 signaling promotes in vivo peripheral nerve regeneration.

Conventional Netrin-1 null knockout mice die within a few hours after birth [102], limiting their utility for studying peripheral nerve regeneration. Instead, Netrin-1 heterozygous mice have been used to study peripheral nerve regeneration with the model of median nerve transection and immediate anastomosis [92]. Although there were no significant differences in the total number of myelinated fibers, axon diameter, fiber diameter and myelin thickness between Netrin-1 heterozygous mice and wild type control mice, the fiber density of regenerating axons was significantly lower in Netrin-1 heterozygous mice. Furthermore, Netrin-1 heterozygous mice showed a much slower functional recovery after median nerve transection and immediate anastomosis and a full functional recovery was never observed [92]. Similar studies have been carried out on Unc5B heterozygous mice and Unc5B heterozygous mice showed a similar regeneration defect to that of the Netrin-1 heterozygous mice [94]. Thus, both reports further confirmed that Netrin-1 is an important molecule in promoting peripheral nerve regeneration.

Schwann cell migration plays a critical role for successful peripheral nerve regeneration in cases of transection injury. Following a peripheral nerve transection, Schwann cells migrate out from both proximal and distal nerve stumps along the newly regenerated blood vessels inside the nerve gap and form a Schwann cell tissue “cable” to direct axon regeneration [107]. Axons are able to regenerate just three hours after transection [108] but Schwann cell migration starts four days later because Schwann cell first need to undergo a process of dedifferentiation before they can begin their migration [107,109]. Therefore, regenerating axons extend randomly into the nerve gap for the first four days after peripheral nerve transection injury due to the lack of Schwann cell guidance. Without the guidance of Schwann cells in a nerve gap, regenerating axons not only elongate with a very low speed (77 µm/day) but also lose their directionality and frequently fail to cross the injury site resulting in no functional recovery [93,108]. After four days, Schwann cells migrate to the front of regenerating axons and then regenerating axons extend their leading processes and attach to migrating Schwann cells to cross the nerve gap. With the guidance of migratory Schwann cells, regenerating axons not only can find their correct path to the distal nerve stump but also increase their growth speed (283 µm/day) for navigating across the nerve bridge [108]. In the zebrafish motor nerve laser transection research model, regenerating axons in the Sox10 mutants, which lack Schwann cells in the peripheral nerve, frequently failed to cross the injury site. Instead, the majority of regenerating axons strayed along aberrant trajectories to invade into adjacent muscle territories around the injury site [93]. Thus, without Schwann cell guidance after peripheral nerve transection injury, regenerating axons lack directionality and travel along ectopic trajectories. Providing axonal scaffolds across the injury site in the Sox10 mutant was insufficient to restore directionality to regenerating axons in the nerve gap, indicating that Schwann cells could produce signaling molecules to direct regenerating axons across the nerve gap.

Schwann cell-derived signals that could attract axon leading processes have not yet been specifically characterized. Neurotrophins such as GDNF, NGF and BDNF have been suggested as candidates for this specific role [110]. Glial cell-derived axon guidance molecules are required to initiate this process during axon extension in neural development. Thus, these developmental signaling pathways could be reactivated during adult peripheral nerve regeneration because adult Schwann cells express Netrin-1 [16], Slit2 and Slit3 [56], Ephrins [19,111] and Semaphorins [20,112] while adult neurons express their receptors [113,114]. Interestingly, regenerating axons also strayed away from their original path onto ectopic trajectories in a zebrafish DCC mutant, which recaptured the phenotype of Sox10 mutant lacking Schwann cells [93]. These findings indicated that Schwann cell derived Netrin-1 could act on the DCC receptor localizing on the growth cone of regenerating axons to direct them towards the distal nerve stump. Indeed, Netrin-1b was found to be expressed in Schwann cells after zebrafish motor nerve transection injury [93]. Thus, Netrin-1 signaling could play an indispensable role in guiding regenerating axons across a nerve gap after peripheral nerve transection injury.

## 9. Future Directions

**Figure 4:**
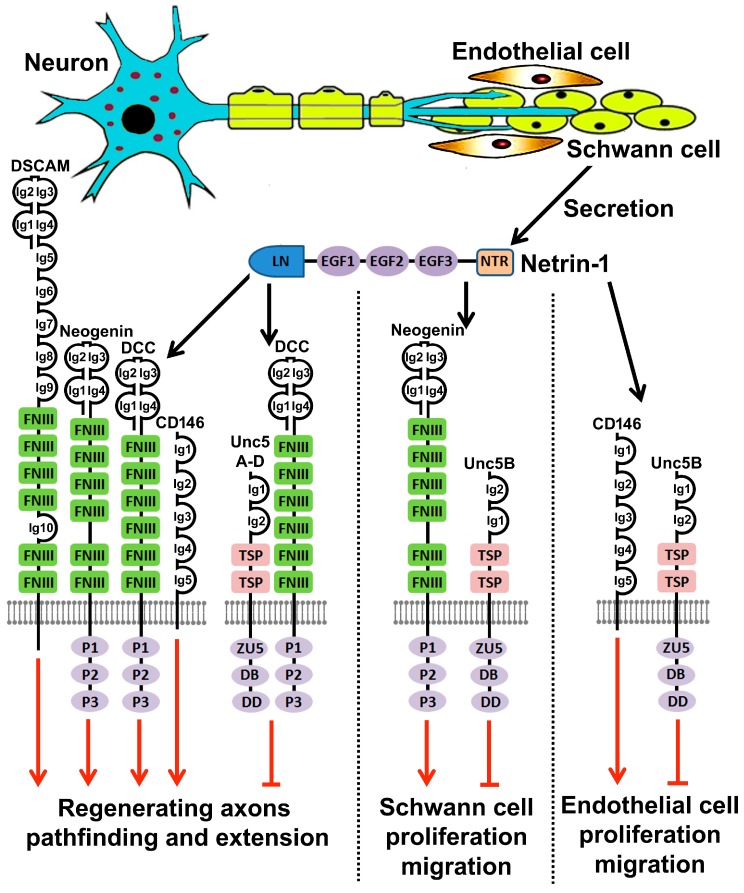
Summary of Netrin-1 function in peripheral nerve regeneration. Schwann cells of the distal nerve stump up-regulate Netrin-1 secretion after peripheral nerve injury. A physiological concentration of Netrin-1 ranging from 50 ng/mL to 150 ng/mL could direct regenerating axons to the distal nerve stump and promote axon extension by interacting with DCC, Neogenin, DSCAM and CD146 on the growth cone of regenerating axons. A physiological concentration of Netrin-1 ranging from 50 ng/mL to 150 ng/mL could also promote Schwann cell and endothelial cell proliferation and migration through the Neogenin receptor and the CD146 receptor, respectively. Unc5A–D are expressed by adult motor and sensory neurons and they could interact with Netrin-1 to slow down the rate of axon extension during regeneration. Unc5B is highly expressed in Schwann cells and endothelial cells, exogenous Netrin-1 with a concentration higher than 500 ng/mL inhibits Schwann cell and endothelial cell proliferation and migration mediated by the Unc5B receptor. Red arrows show Netrin-1 attractive signaling, red T-bars show Netrin-1 repulsive signaling.

Recent studies have pointed to an important role of Netrin-1 in peripheral nerve regeneration (Figure 4). However, the in vivo functions of Netrin-1 still require further investigation. Conventional Netrin-1 and Netrin-1 receptor gene knockout mice die in the embryonic stage or postnatally, which prevents their use in studying the function of Netrin-1 signaling in nerve regeneration. Recently, conditional Netrin-1 [115] and Netrin-1 receptor [23,116,117,118] gene knockout mouse models have been generated by different research groups, but these research groups are often working in the research field of nervous system development and tumourigenesis. Collaboration between these research groups with other groups who are interesting in studying the role of Netrin-1 signaling in nerve regeneration will help us understand a better in vivo role of Netrin-1 signaling in these regenerative processes. A major obstacle for peripheral nerve repair is the misdirection of regenerating axons in the nerve gap after peripheral nerve transection injury [109,119,120,121]. Netrin-1 has a strong ability to attract axon extension towards its source. It will be interesting to study how Netrin-1 could promote regenerating axons targeting the distal nerve stump with high accuracy.

DSCAM and CD146 are recently identified Netrin-1 receptors. Their expression patterns in the adult peripheral nervous system have not yet been fully studied. Therefore, their function in nerve regeneration is currently unknown. Angiogenesis is an important process for peripheral nerve regeneration after transection injury, newly formed blood vessels not only provide oxygen and supply nutrition to cells within the nerve bridge, but also function as a substrate for Schwann cells migrating into the nerve gap [107]. CD146 is highly expressed on endothelial cells and Netrin-1 can promote angiogenesis though CD146 [23]. It will be interesting to study if Netrin-1 could promote in vivo blood vessel regeneration in the nerve gap through CD146.

The attractive function of Netrin-1 could be utilized to promote peripheral regeneration at a physiological concentration ranging from 50 ng/mL to 150 ng/mL. However, the predominant expression of Unc5A–D in adult neurons will activate Netrin-1 repulsive signaling to slow down the rate of axon regeneration. Recent structural studies have revealed that the Netrin-1 EGF2 domain is required for Unc5A–D binding [44,50]. However, mutation of key amino acid residues in the EGF2 domain not only prevents Unc5A–D binding but also abolishes Netrin-1 attractive signaling through DCC binding [44]. Furthermore, it is currently unclear if the Netrin-1 EGF2 domain is required for CD146 and DSCAM binding. Thus, more Netrin-1/receptor crystal structure studies are required in order to design a Netrin-1 modified molecule that binds to distinct receptors. Interestingly, the anti-Netrin-1 antibody generated by Grandin et al blocks Netrin-1/Unc5B interaction but not Netrin-1/DCC interaction [50]. This antibody binds to an amino acid on the Netrin-1 EGF2 domain, which is not one of the key amino acid residues identified by the structure studies for Netrin-1 binding to DCC and Unc5B [44,50]. It will be interesting to see if this antibody could perhaps inhibit Netrin-1 repulsive signaling when an attractive signaling is needed for therapeutic purposes, for example, to promote nerve regeneration.

## Figures and Tables

**Table 1:** Netrin-1 and Netrin-1 receptor expression in intact and injured adult rodent spinal cord.

	Gray Matter		White Matter	Injury Response
	Dorsal	Ventral		
Netrin-1	Oligodendrocyte (+++) Interneurons (++)	Oligodendrocyte (+++) Motorneurons (++)	Oligodendrocyte (+++)	Down-regulated
DCC	Interneurons (+)	Motorneurons (++)	Glia (+)	Down-regulated
Neogenin	Interneurons (+)	Motorneurons (++)	Glia (+)	Down-regulated
Unc5A	Interneurons (+)	Motorneurons (+++)	Glia (+)	Down-regulated
Unc5B	Interneurons (+++)	Motorneurons (+++)	Glia (++)	Down-regulated
Unc5C	Interneurons (++)	Motorneurons (+++)	Glia (+)	Down-regulated
Unc5D	Interneurons (+)	Motorneurons (+)	Glia (+)	Down-regulated

(+): low level of expression, (++): medium level of expression, (+++): high level of expression.
